# Workflow for Fluorescence-Targeted Lamella Milling From Vitrified Cells With a Coincident Fluorescence, Electron, and Ion Beam Microscope

**DOI:** 10.21769/BioProtoc.5390

**Published:** 2025-07-20

**Authors:** Elise G. Perton, Daan B. Boltje, Arjen J. Jakobi, Jacob P. Hoogenboom

**Affiliations:** 1 Department of Imaging Physics, Delft University of Technology, Delft, The Netherlands; 2 Kavli Institute of Nanoscience, Delft University of Technology, Delft, The Netherlands; 3 Delmic B.V., Delft, The Netherlands

**Keywords:** Cryo LM-FIB-SEM, Cryo-SECOM, CLEM, Cryo-ET, Coincident microscope, Fluorescence targeted milling, FIB-SEM, Quality control, Lamella thickness control, ENZEL system, TEM

## Abstract

Cryo-electron tomography (cryo-ET) is the main technique to image the structure of biological macromolecules inside their cellular environment. The samples for cryo-ET must be thinner than 200 nm, which is not compatible with micron-sized cells. A focused ion beam (FIB), in conjunction with a scanning electron microscope (SEM) to navigate the sample, can be used to ablate material from vitrified cells such that a thin lamella remains. However, the preparation of lamellae with a FIB-SEM is blind to the location of specific cellular structures and biomolecules. Furthermore, the thickness and uniformity of lamella, while crucial for high-quality tomograms, cannot be established accurately with the FIB-SEM. These limitations strongly affect the success rate for cryo-ET on FIB-milled lamellae and thereby the total throughput of the workflow. To mitigate these problems, a coincident light, electron, and ion beam cryo-microscope was developed by retrofitting a fluorescence microscope, cryogenic microcooler, and piezo stage on a FIB-SEM. The fluorescence of molecules of interest can be monitored in real time while milling to ensure the final lamella contains the structure of interest. In addition, reflected light microscopy can be used for thickness and quality control of the lamella. In this protocol, we will describe how the coincident microscope can be used to prepare lamellae from vitrified cells.

Key features

• Step-by-step protocol for fluorescence-guided FIB-milling with a coincident three-beam cryogenic microscope as described in [1].

• Details about sample loading and unloading, as well as the lamella milling workflow with graphical explanations.

• Quality control of lamella, including thickness, uniformity, and ice contamination.

## Background

To obtain a full mechanistic understanding of how biological macromolecular complexes execute their function, their structures must be imaged within their cellular context. Cryogenic electron tomography (cryo-ET), combined with subtomogram averaging, allows determination of 3D structures in situ at high resolution [2]. The cryo-ET workflow includes growing cells on a transmission electron microscopy (TEM) grid, flash-freezing the grid, thinning the sample, and acquiring a tilt series in the TEM [3]. A focused ion beam (FIB) guided by a scanning electron microscope (SEM) is used to remove material from the sample such that a thin slice known as the lamella remains. This lamella can only be used to obtain a high-quality 3D reconstruction of the structure of interest if the lamella has a uniform thickness below 200 nm, is free from contamination such as crystalline ice, and the structure of interest is present within the lamella [2].

To determine the location of the target biomolecule inside the sample, the biomolecule can be fluorescently labeled, and the sample can be imaged with a cryogenic fluorescence microscope (cryo-FM) before and after FIB milling [4,5]. However, the extra handling steps involved in cryo-FM increase the risk of devitrification and contamination of the sample. To overcome this, integrated systems were developed where the stage can move between FM and FIB locations [6–8]. Yet, the correlation accuracy between the FIB and FM and the transfer time between FIB milling and FM imaging limit the precision and throughput of lamella fabrication.

By combining a SEM, FIB, and FM with a single coincident point at cryogenic conditions, fluorescence intensity can be tracked while FIB milling. We previously built a coincident FM-FIB-SEM by combining an existing FIB-SEM with the ENZEL system (Delmic B.V.), composed of a cryogenic microcooler, an inverted widefield FM, and a positioning stage [1]. The objective lens of the FM in ENZEL has a higher numerical aperture (NA) and shorter working distance compared to other three-beam coincident systems, such as ELI [9] and Arctis Cryo-Plasma-FIB (Thermo Fisher Scientific), leading to improved resolution and light-gathering capabilities, and therefore superior quality FM. In addition, the FM in the Arctis cannot be operated simultaneously with the FIB to obtain real-time information on the molecule of interest, in contrast to the ENZEL system. With the light microscope (LM) in reflected light microscopy (RLM) mode, the sample can be screened for the presence of ice crystals [10]. The thickness and uniformity of the lamella can also be determined with RLM, as well as the integrity of the protective platinum coat [10].

In this protocol, we describe how to use the coincident system for FM-guided lamella milling and quality control of lamella starting from a vitrified, clipped grid with cells. We refer to [11–13] for more information on preparing vitrified grids with cells. First, we describe how to load the sample. We use a custom glovebox to minimize contamination by crystalline ice during sample loading. Second, we describe how to mill lamellae while making optimal use of the additional information provided by the LM. We finish by describing how to unload the sample. The protocol is directly applicable to our ENZEL system at TU Delft—as TU Delft is at present the only location with this custom glovebox—and can be applied with some modifications in the (un)loading process to the two other ENZEL systems (at the Centre for Microscopy and Microanalysis of the University of Queensland and at the Dahlberg research group at Stanford University and SLAC National Accelerator Laboratory [14,15]). The ENZEL system can be used to increase the throughput of lamella fabrication and is especially useful if the biological structure of interest is sparse (i.e., only present in a fraction of cells and/or only present in a limited region of the cell).

## Materials and reagents

**Biological materials**

1. Vitrified cells containing fluorescently tagged biomolecules of interest

*Note: The figures in this protocol were prepared using HeLa cells expressing microtubule-associated protein 1A/1B light chain 3B (LC3B) tandem tagged with mRFP and GFP [16]. These cells were also used in our previous publications about the coincident system [1,10] and kindly gifted by Fulvio Reggiori from the University of Groningen, Netherlands.*

**Reagents**

1. Liquid nitrogen

**Laboratory supplies**

1. AutoGrids composed of:

a. Cryo-EM grids (Quantifoil R2/2 Au 200 mesh were used to prepare the figures in this protocol, but other grid types can be used as well)

b. C-clip (Thermo Fisher Scientific, catalog number: 1036171)

c. C-clip ring (Thermo Fisher Scientific, catalog number: 1036173)

2. Coverslips Schott D263M glass with ITO coating, diameter 3.4 mm, thickness 0.17 mm (22 × 22 × 0.17 mm cover glass with ITO from Optics Balzers, cut by Lasertec)

3. Carbon-tipped tweezers (ideal-tek, catalog number: 2ACFR.SA)

4. Fine tweezers (Dumont, catalog number: 0102-P-PO-1)

5. Large tweezers (Knipex, catalog number: 92 61 02)

6. Criterium 2 mm mechanical pencil to grip cryo grid boxes, referred to as gridbox pen (BIC, catalog number: 3270220029748)

7. Torlon hex key (Delmic B.V., delivered with system)

8. Hot plate (Cole Palmer)

9. Storage dewar (MVE Biological Solutions)

10. Thermos flask (Ikea, catalog number: 604.153.51)

11. Liquid nitrogen bath (Hendi, catalog number: 8711369806432)

12. Falcon tube with rope attached; a small hole is drilled through the 50 mL Falcon tube near the 45-mark to attach a 2 mm rope that facilitates the transfer of the tube from and to liquid nitrogen (Falcon, catalog number: 352070)

13. Torque limiter set to 10.15 cN×m (Bestool Kanon, catalog number: CN15LTDK-H)

## Equipment

1. Dualbeam FIB-SEM system; for this protocol, we used a Helios NanoLab G3 UC (Thermo Fisher Scientific)

2. ENZEL base system (Delmic B.V.):

a. High vacuum door assembly

b. Integrated epifluorescent microscope

c. Cryogenic sample transfer loadlock and sample shuttle

d. Joule Thompson microcooler, including CryoLab control electronics, CryoVision control software, and Lakeshore Model 355 temperature controller (Demcon-kryoz)

e. Piezo positioning stages for cryogenic cooler (SmarAct GmbH)

f. Piezo positioning stages for optical objective lens (SmarAct GmbH)

g. Turbo molecular pump for loadlock (Pfeiffer Vacuum)

3. Cryogenic chamber shield (Microscopy Solutions, NRE quote MS0204)

4. Glovebox system (i.e., KIYON) with 85 mm diameter hole in bottom, including swiveldoor to close

5. Parts to interface transfer module to glovebox (listed in Table S1 and 3D STEP files in [18]), assembled as shown in Figures S1 and S2 and described in Table S1

*Note: The custom interface assembly without glovebox or transfer vessel parts, as listed in Table S1 and 3D STEP files, can be found in [18].*

## Software and datasets

1. CryoVision (version 0.2.3.0, Demcon-kryoz)

2. xT Microscope Control (version 10.1.7)

3. iFast Developer’s Kit (version 5.1.10.2037)

4. Odemis [17] (version 3.3.0-174-g9355cace9)

5. Odemis plugins [18]

6. Intensity fluorescence monitor IFM-Monitor.ipynb [18]

7. iFast script LamellaMillingCommands.xrml [18]

## Procedure

**Caution:** Wear goggles and cryogenic gloves when handling liquid nitrogen. Ensure adequate ventilation.

For each step, an indication of the time it takes to complete the step is included. This is only an approximation, as the duration will depend on the experience of the user and the number of features.

**A. Sample loading**

**Figure 1.**
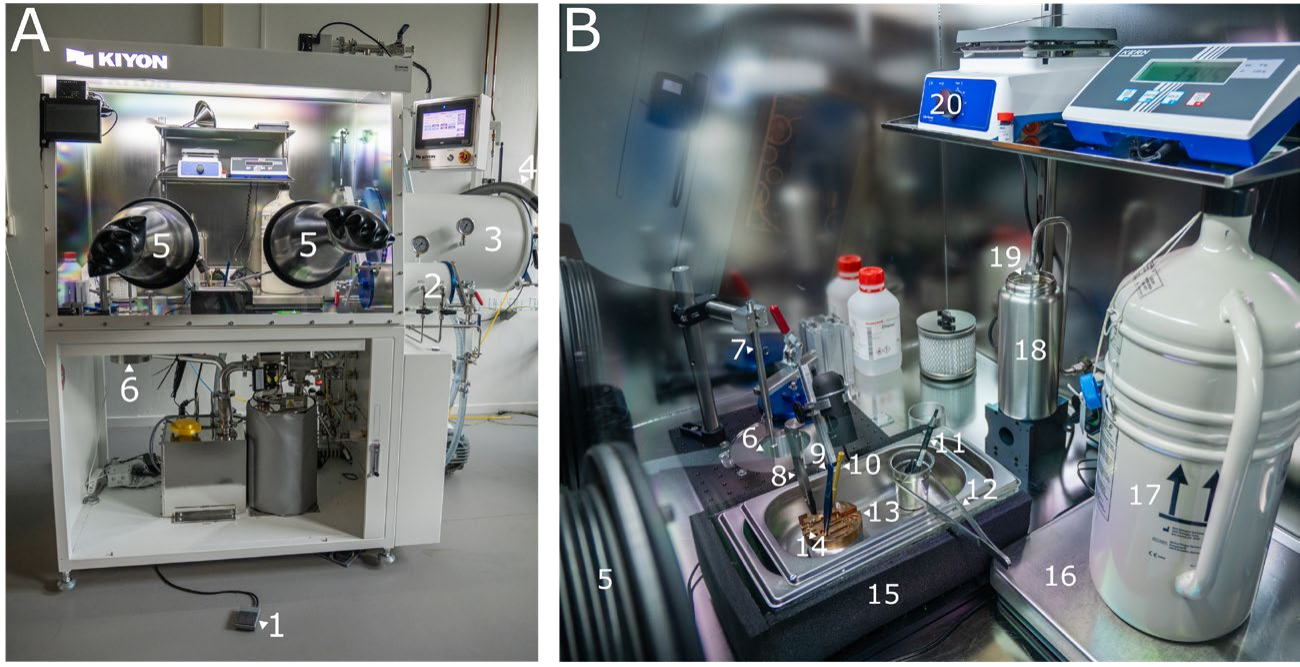
Overview of the glovebox. (A) Outside view of the glovebox. (B) Inside view of the glovebox. The numbering of components in A corresponds to the numbering in B. 1: Foot pedal. 2: Small airlock. 3: Large airlock. 4: Liquid nitrogen hose. 5: Glove. 6: Transfer module. 7: Pickup rod. 8: Carbon-tipped tweezers. 9: Fine tweezers. 10: Hex key. 11: Gridbox pen. 12: Large tweezers. 13: Cold table. 14: Shuttle. 15: Sample preparation bath. 16: Scale. 17: Storage dewar. 18: Thermos flask. 19: Liquid nitrogen outlet. 20: Hot plate.

**Figure 2.**
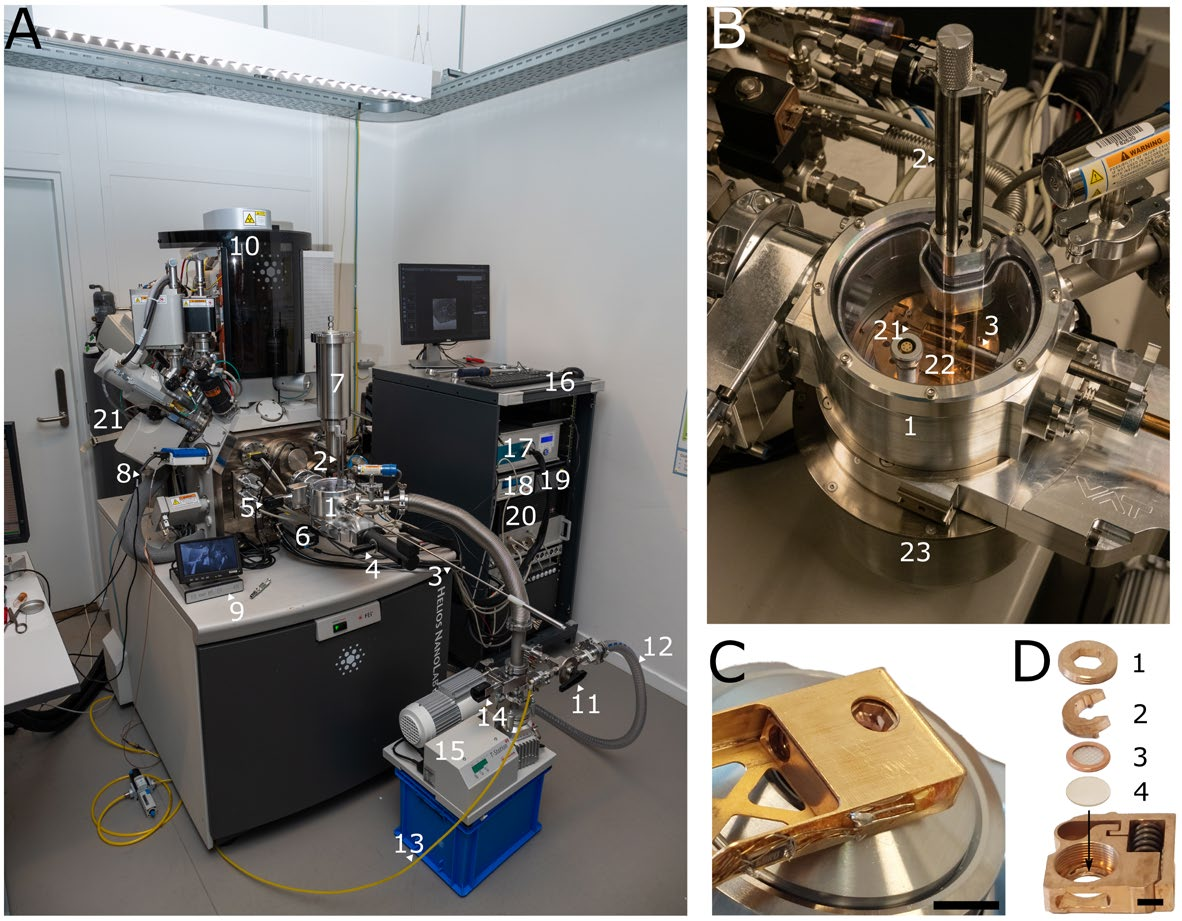
Overview of the microscope and shuttle. (A) ENZEL system retrofitted on a Helios Nanolab 650 dual-beam FIB-SEM. 1: Load-lock chamber. 2: Vertical transfer rods. 3: Transfer rod. 4: Gate valve between the load-lock chamber and transfer module. 5: Gate valve between the load-lock chamber and the SEM chamber. 6: LM camera. 7: Cryogenic chamber shield dewar. 8: Small dewar. 9: Debian infrared camera module. 10: Helios Nanolab G3 UC. 11: Three-way valve. 12: Roughing pump hose. 13: Dry nitrogen tubing. 14: Gate valve between the turbo-molecular pump and the load-lock chamber. 15: Turbo-molecular pump. 16: Electronics rack. 17: Microcooler electronics. 18: Lakeshore temperature controller. 19: Pressure monitor. 20: Linux microscope computer with Odemis. (B) Load-lock chamber of ENZEL during loading. 1: Load-lock chamber. 2: Vertical transfer rods. 3: Transfer rod. 21: Shuttle. 22: Cold table. 23: Transfer module. (C) Shuttle inside the shuttle holder above objective lens. Scale bar 5 mm. Adapted from Figure 3b of the original paper describing the system [1], used under CC BY 4.0. (D) Shuttle with 1: screw, 2: spacer ring, 3: AutoGrid, 4: coverslip. Scale bar 2 mm. Adapted from Figure 3c of the original paper describing the system [1], used under CC BY 4.0.

This protocol assumes that your sample is located in a liquid nitrogen–filled storage dewar inside the glovebox. To facilitate the understanding of this protocol, annotated photographs of the glovebox and the ENZEL base system retrofitted on a dual-beam FIB-SEM system are included. We refer to Figure 1 for an overview of the components and items used within the glovebox. Figure 2 shows the exterior of the microscope as well as the shuttle design. For a more detailed description of the internal design of the coincident system, we refer to the original paper describing the coincident system [1].

1. Cool down cryogenic shields (5 min).

a. Fill the cryogenic chamber shield dewar with liquid nitrogen until the boil-off prevents further filling.

b. Fill the small dewar mounted at the back of the chamber with liquid nitrogen until the boil-off prevents further filling.

c. When the cryogenic chamber shield dewar is thermalized, fill it up completely and put the lid back on.

d. When the small dewar mounted at the back is thermalized, fill it up completely and put the lid back on.

2. Cool down microcooler (1 min to set up, up to 2 h to cool down).

a. Start CryoVision and connect to the cooler by clicking the *power* button in the top right corner.

b. To start logging the temperature, go to *Functions → Start logging*.

c. Set the temperature control to 90 Kelvin and click *Start temperature control*.

d. In the *Chamber* tab of the Odemis graphical user interface, turn on *Sample heater* if it was turned off.

e. Wait for the cooler to reach the set point. This takes up to 2 h. Once the setpoint is reached, a sample can be loaded.

3. Prepare the glovebox (5 min).

a. Open the liquid nitrogen dewar and turn on the light inside the glovebox.

b. Use the foot pedal to fill the thermos flask inside the glovebox with liquid nitrogen.

c. Insert your hands into the gloves in the glovebox. Fill the sample preparation bath and the transfer module with liquid nitrogen. If the cold table is not placed inside the preparation bath, do so. If the pickup rod is not placed inside the transfer module, do so.

d. Wait for the cold table to thermalize, as can be seen by a strong decrease in the bubbles. Add liquid nitrogen to the sample preparation bath and the transfer module as needed to compensate for boil-off.

4. Mount sample in glovebox (10 min).

a. Take the tools (fine tweezers, carbon-tipped tweezers, gridbox pen, and hex key) from the hotplate and slot them in the cold table to precool their tips. Position the large tweezers in the sample preparation bath. Wait for the tools to thermalize.

b. Open the shuttle cover with the large tweezers.

c. Use the hex key to remove the screw inside the shuttle and place it in an empty recess on the cold table.

d. Use the fine tweezers to take out the spacer ring and place it in the same recess as the screw.

e. Take the Falcon tube with your gridbox from the storage dewar and place it in the metal tea sieve basket inside the bath.

f. Place the gridbox into the fitting recess in the cold table using the big tweezers. Remove the lid with the gridbox pen.

g. Use the carbon-tipped tweezers to take an AutoGrid from the gridbox and place it in an empty recess on the cold table.

h. Re-cap the gridbox with the gridbox pen and place it into the Falcon tube. Place the Falcon tube into the storage dewar.

i. Pick up the AutoGrid and place it inside the shuttle using the fine tweezers. Ensure that the grid is placed with the cells facing upward (grey side of grid), so that the flat side of the AutoGrid is facing upward.

j. Insert one fine tweezer tip into one of the small holes in the spacer ring and place the spacer ring in the shuttle with the opening facing the FIB access hole.

k. Use the hex key to place the screw on top of the shuttle and carefully screw it hand tight.

l. Check that the top of the screw is below the shuttle surface. If this is the case, all parts are mounted correctly.

m. Close the shuttle cover.

n. Place all tools on the hot plate set to 70 °C and turn on the hot plate timer (4 h).

5. Load cold table in transfer module (3 min).

a. Screw the pre-cooled pickup rod into the cold table.

b. Use the pickup rod to transfer the cold table to the transfer module. Insert the thinner part of the pickup rod in the aluminum guiding block.

c. Lower the cold table into the transfer module until it sits in the mount at the bottom.

d. Unscrew the pickup rod and remove it from the transfer module.

e. Close the transfer module valve.

f. Remove your hands from the glovebox and open the valve on the side of the transfer module to relieve the pressure inside.

g. Undock the transfer module from the glovebox by rotating the transfer module (counterclockwise, looking from the top).

h. Put the lid on the transfer module.

i. Transport the transfer module to the ENZEL.

6. Load cold table in ENZEL (5 min).

a. In the *Chamber tab* of the Odemis graphical user interface, click *Loading* to move the sample positioning stage to the loading position.

b. Check that the turbo molecular pump is running.

c. Remove the lid from the transfer module.

d. Align the transfer module with the load-lock chamber and rotate clockwise (looking from the top) until the spring pin clicks and locks the rotation.

e. If the valve on the side of the transfer module is closed, open it to relieve the pressure from the liquid nitrogen boil-off.

f. Check that the gate valve between the load-lock chamber and SEM chamber is closed.

g. Close the gate valve between the load-lock chamber and turbo molecular pump.

h. Partially open the gate valve between the load-lock chamber and transfer module.

i. Vent the load-lock chamber by letting in dry nitrogen via the three-way valve.

j. Close the three-way valve when the load-lock chamber reaches atmospheric pressure (hissing increases from the pressure relief valve at the side of the transfer module).

k. Open the gate valve between the load-lock chamber and transfer module.

l. Lower the vertical transfer rod tips into the liquid nitrogen to pre-cool them.

m. Unfasten the transfer rod rest hook.

n. A brief increase in the hissing noise from the pressure relief valve indicates that the transfer rod tips are thermalized. Engage the two vertical feedthrough guiding rods and screw the third rod into the cold table.

o. Lift the cold table fully up from the transfer module into the load-lock chamber.

p. Close the gate valve between the load-lock chamber and transfer module.

q. Screw the transfer rod tip into the lower hole in the cold table using the transfer rod to pre-cool the transfer rod tip.

*Note: Do not force this step, as the thread is fragile.*

r. Screw the vertical transfer rod locking screw tightly.

s. Slowly open the three-way valve toward the roughing pump while monitoring the liquid nitrogen evaporation.

*Note: Avoid aggressive pumping as this will solidify the liquid nitrogen.*

t. When all liquid nitrogen has evaporated, open the three-way valve to the roughing pump fully and evacuate the load-lock chamber to 0.1 mbar.

u. Close the three-way valve and open the gate valve to the turbo molecular pump. Monitor the pressure. When it goes out of range, wait 30 s.

v. While waiting, enable the infrared CCD camera in xT Microscope Control and switch on the Debian camera module.

w. Open the gate valve between the load-lock chamber and the SEM. In the bottom left corner of xT Microscope Control, monitor the *Chamber Pressure*. It should not exceed 3×10^-6^ mbar. If it does, close the gate valve.

7. Load shuttle in ENZEL (2 min).

a. In the *Chamber tab* of the Odemis graphical user interface, check that the *Loading* button lights up green (the sample positioning stage is in loading position). If not, click the *Loading* button.

b. Attach the torque limiter at the end of the transfer rod.

c. Unscrew the transfer rod tip from the cold table and screw it into the shuttle.

d. Carefully retract the transfer rod to take the shuttle out of the cold table.

e. Lower the cold table slightly.

f. Insert the transfer rod 30 cm while keeping it level.

g. Lower the end of the transfer rod to lift the tip of the rod and insert a bit further. The rod should be in the field of view of the Deban infrared CCD camera.

h. Carefully move the transfer rod further and slide the shuttle into the holder. It is fully inserted when there is no gap between the copper and white plastic parts of the transfer rod.

i. Unscrew the transfer rod from the shuttle using the torque limiter. The shuttle shield will rotate itself around the holder.

j. Rotate the shield around the holder and carefully retract the transfer rod 30 cm while moving your hand down slightly to lift up the front end of the rod.

k. Level the transfer rod and retract until the tip is centered in the load-lock chamber.

l. Close the gate valve to the SEM chamber and secure rest hook.

8. Unload the cold table in ENZEL (3 min).

a. Loosen the vertical transfer rod locking screw slightly.

b. Check that the pressure relief valve on the side of the transfer module is opened slightly.

c. Close the gate valve to the turbo molecular pump.

d. Partially open the gate valve between the load-lock chamber and transfer module.

e. Vent the load-lock chamber by letting in dry nitrogen via the three-way valve.

f. When the load-lock chamber reaches atmospheric pressure (the big gate valve makes a small clicking noise), quickly open the gate valve between the load-lock chamber and transfer module fully and submerge the cold table into liquid nitrogen.

g. Close the three-way valve.

h. Check that the cold table is properly seated inside the transfer module.

i. Unscrew the third rod from the cold table and lift the two guiding rods out of the cold table. Lift out the rods from the transfer module.

j. Close the gate valve between the load-lock chamber and transfer module.

k. Stop the turbo molecular pump to minimize vibrations during the next steps.

l. Open the roughing pump to the three-way valve to evacuate the load-lock chamber.

m. Lift the spring pin and rotate the transfer module (counterclockwise, looking from the top) to undock it from the load-lock chamber.

n. Put the lid on the transfer module and close the pressure relief valve on the side.

o. Transfer the transfer module to the glovebox.

9. Unload cold table in glovebox (5 min).

a. Remove the lid of the transfer module.

b. Align the transfer module with the glovebox bayonet interface and rotate the transfer module (clockwise, looking from the top).

c. Open the nitrogen valve to flush the transfer module with dry nitrogen.

d. Open the valve to the roughing pump and wait 15 s.

e. Close the dry nitrogen valve and wait 10 s.

f. Close the valve to the roughing pump.

g. Insert your hands into the gloves of the glovebox and open the transfer module valve.

h. Insert the transfer rod in the guiding mechanism. Lower the rod and screw it in the cold table.

i. Lift out the cold table and transfer it to the liquid nitrogen bath.

j. Unscrew the pickup rod and store it in the guiding mechanism.

k. Remove your hands from the glovebox.

l. Switch on the timer for the hairdryer to avoid condensation and ice forming on the transfer module.

m. Turn off the light and close the liquid nitrogen dewar.

**B. Lamella milling**

**Figure 3.**
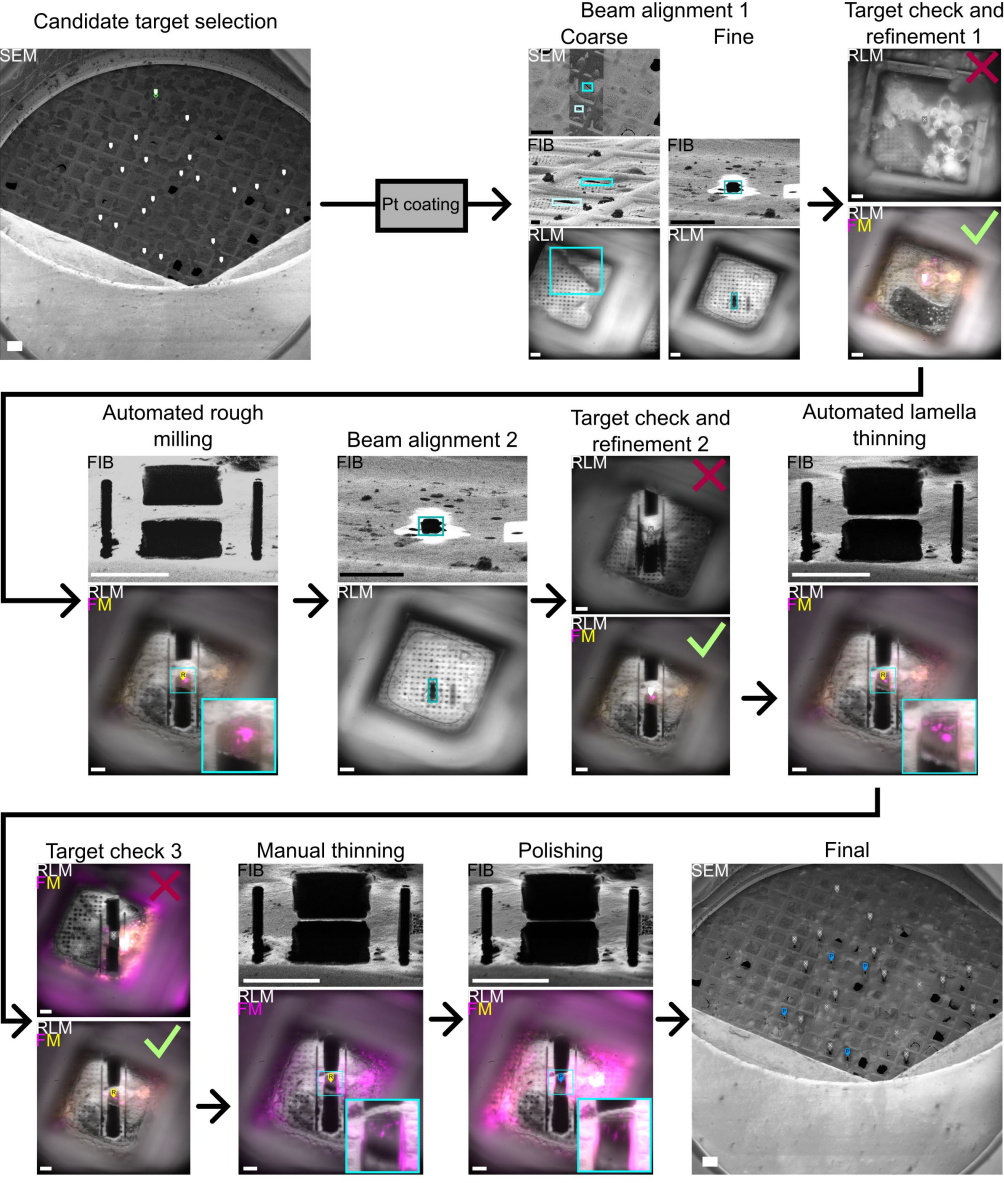
Lamella milling workflow. Candidate locations for lamella milling are identified in a low-magnification SEM image. After platinum (Pt) coating, SEM, FIB, and RLM beams are first aligned coarsely using features present in all views (blue boxes in Beam alignment 1, Coarse), and then finely by milling an alignment pattern using the FIB (blue boxes in Beam alignment 1, Fine). All candidate targets are checked with RLM for ice and grid foil integrity, and with FM for the presence and location of fluorescence. Based on this, a feature is discarded (top) or its position is updated (bottom). All active features are automatically rough-milled. After this, the FIB current is decreased, and FIB and RLM are re-aligned using an alignment pattern. Then, all rough-milled targets are checked with RLM for grid foil integrity and with FM for the presence and location of fluorescence. Based on this, a feature is discarded (top) or it is set to active, and its position is updated (bottom). All active features are automatically thinned to ~2 μm. Then, all thinned features are checked with RLM for grid foil integrity and with FM for the presence and location of fluorescence. Based on this, a feature may be discarded (top). Note that the fluorescence channels in this image have been augmented to provide an example of a lamella without fluorescence. Lamellae are then further thinned and polished manually while keeping track of the fluorescence and RLM for Pt coating integrity. Before unloading, an overview of the grid with all polished lamella is acquired with the SEM. Scale bars of SEM images are 100 μm, FIB 10 μm, and RFLM/FM 10 μm.

**Figure 4.**
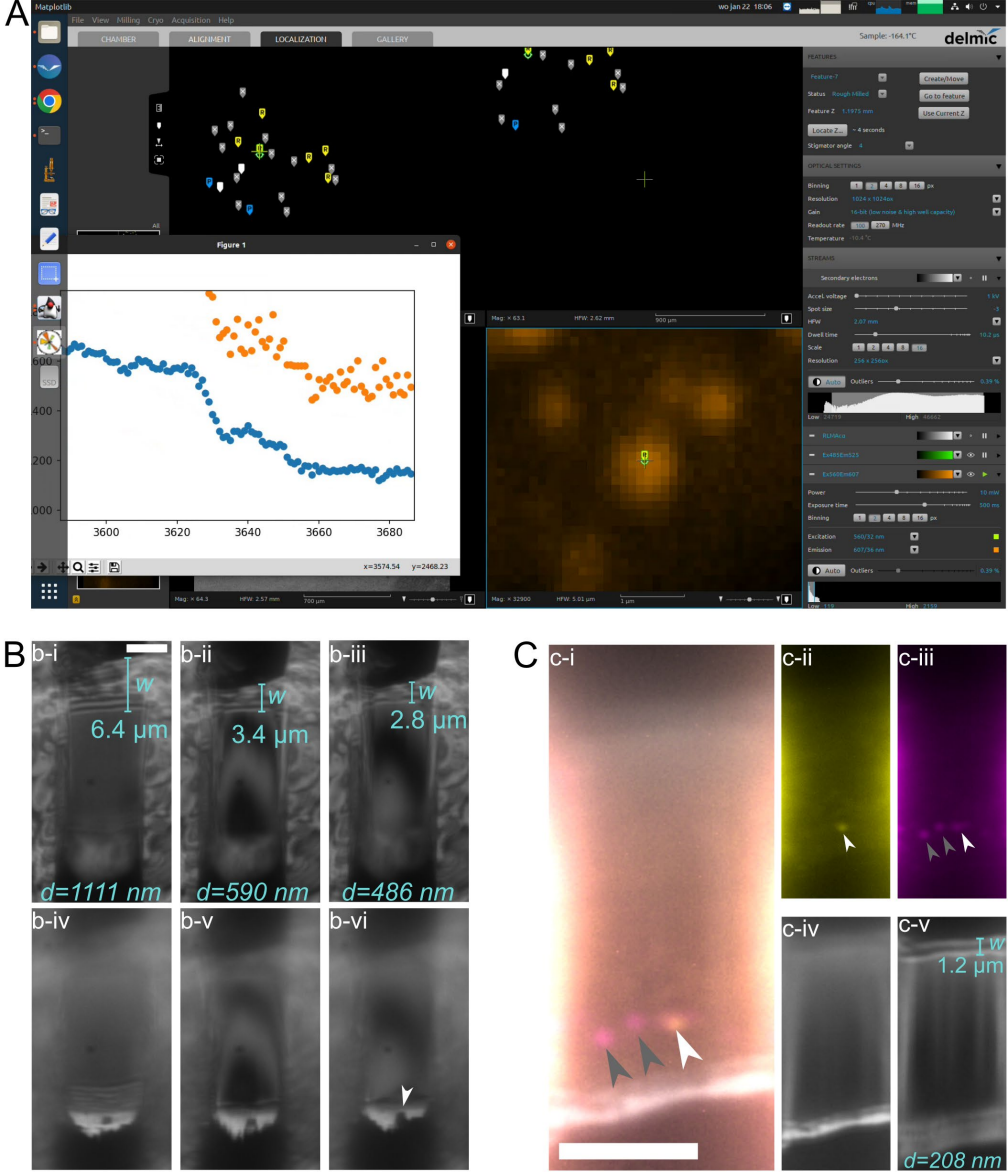
Usage of reflective light microscopy (RLM) and fluorescence microscopy (FM) during manual thinning for lamella thickness and quality control. (A) Target fluorescence inside the lamella is tracked in real time during the milling process. Screenshot of the Linux PC running Odemis (main window) and the IFM-monitor (popup plot). (B) With RLM, the thickness of the lamella (i-iii) and the integrity of the platinum (Pt) coat (iv-vi) can be monitored. The lamella thickness d can be determined by measuring the width w of the interference pattern at the lower wedge of the lamella (upper side in image) (i-iii), and using d = sin(θ)·w = 0.174w, given that the milling angle is fixed to 10° [10]. Platinum (Pt) is visible as a bright white line at the top of the lamella (iv-vi). We see that the Pt is running out locally (iv, white arrow), preventing thinning of the lamella beyond ~490 nm (iii). (C) Lamella containing fluorescent features of interest (i-iii, arrowheads) and Pt layer and lamella wedge in RLM (iv-v). i: Overlay of two FM channels (GFP, yellow; mRFP, magenta) and RLM. White arrowhead indicates autophagosome, and grey arrowheads indicate autolysosomes. ii: GFP channel of i. iii: mRFP channel of i. iv: Pt is visible as a bright white line in RLM. v: The lamella is ~230 nm thick, as determined by the width of its interference pattern. All scale bars are 5 μm, and all lamellae are from mRFP-GFP-LC3 HeLa cells.

Steps B2–11 are depicted in Figure 3. For Figures 3 and 4, HeLa cells expressing Mrfp-GFP-LC3B were used, as in our previous publications about the coincident system [1,10]. Colocalization of Mrfp and GFP indicates autophagosomes, while Mrfp alone indicates autolysosomes.

1. Set up the software and move the sample positioning stage (5 min).

a. In xT Microscope Control, press *Wake up* (under *System*) to turn on the FIB and SEM.

b. In xT Microscope Control, set FIB and SEM *Scan Rotation* (under *Scan Rotation*) to 180°.

c. In the *Chamber tab* of the Odemis graphical user interface, click *3Beams*. A pop-up will appear. Check that the transfer rod is removed and click *Load*.

d. Wait until the sample positioning stage has been referenced. Five seconds later, cancel the move by clicking the *Cancel* button in the Odemis graphical user interface. This step is required due to a bug in Odemis.

e. Again, click *3Beams* in the Odemis graphical user interface. Wait for it to finish.

f. Switch to the *Alignment tab* of the Odemis graphical user interface and press *Move to latest custom alignment*.

g. Switch to the *Localization tab*.

h. If you need to set up the fluorescent streams (e.g., because the Odemis graphical user interface has restarted), click *Cryo* → *Set-up Streams* in the top bar. Remove the original *Filtered Color 1* stream as well as any other fluorescent streams you do not need for imaging.

*Note: You need two streams per wavelength: one for live imaging and one for image acquisition.*

2. Create SEM overview image and select candidate locations for lamella fabrication (10 min).

a. In the *Localization tab* of the Odemis graphical user interface, enable the *RLMAcq* stream.

b. Hover the mouse above the RLM stream. Hold the right mouse button and move the mouse up or down to get the grid foil into focus.

c. Select the *Secondary electrons* stream and set the following settings: *Dwell time 10.2 μs, Scale 16 (256* × *256), HFW 2 mm*.

d. In xT Microscope Control, set the SEM stream to 1 kV, 0.10 nA, magnification 100×. In the Odemis graphical user interface, acquire a SEM image by unpausing the stream. Pause once the image has updated.

f. Double-click on the grid center in the SEM image to center the grid in the SEM field of view.

g. Select the *Secondary electrons* stream and adjust the *Scale* to *2 (2048 × 2048)*

h. Acquire a SEM image by unpausing the stream. Pause once the image has updated.

i. Press F5 to show the SEM image in full frame and look for candidate cells for lamella fabrication. To create a feature for a candidate cell, click *Create/Move* (under *Features*) and click on the target location on the SEM image. Ideal candidate cells are located at the center of the grid square, as the feature needs to be in the middle 40 μm of the grid square to be accessible to both FIB and TEM tomography. The TEM used downstream can constrain which grid squares are usable for tomography, as not all locations on the grid allow tilting from -60° to 60°. Check this with your TEM operator. Tears in the grid foil can be visible in the SEM overview image. Avoid cells in grid squares with broken foil. Crystalline ice contamination on the top (side with cells) of the grid may be visible in the SEM overview image. In this case, avoid cells in grid squares with this ice contamination.

j. Click *File* → *Save Snapshot As* to save the SEM overview image.

k. Press F5 to leave full frame mode.

3. Platinum (Pt) coat the grid using the gas injection system (GIS) (5 min). The Pt layer protects the vitrified cells from the FIB and reduces curtaining artefacts.

a. Go to the *Chamber tab* of the Odemis graphical user interface and press *Coating*.

b. In xT Microscope Control, enable the infrared CCD camera and wait until the sample positioning stage has reached the coating position.

c. In the Odemis graphical user interface, click *Cryo* → *Move stage…* in the top bar. A pop-up *Move sample stage* will appear.

d. Set the value of *dz* to 200 μm.

e. Click the *Z-*button three times to lower the stage 600 μm. At the same time, look at the infrared CCD camera stream in xT Microscope Control to check that the stage moves down.

f. In xT Microscope Control, click *FEI* (top left) → *Service Tools* → *Positioning*. Inside the window *Position test dialog* that opens, click the *Z to FWD* button. Close the window.

g. In xT Microscope Control, go to *Patterning Control* → *Gas Injection* and click the *Insert* checkbox for the platinum precursor.

h. In the popup that appears, click *Yes* to confirm you want to insert the GIS needle into the chamber.

i. In the Odemis graphical user interface, click the *Z+* button six times to move the stage up 1,200 μm. Simultaneously monitor the sample holder temperature to verify that the GIS needle does not touch the sample holder. If the temperature does rise, click the *Z-*button to lower the stage 200 μm. The sample is now in the right position for Pt coating.

j. In xT Microscope Control, click the *Closed* button of the platinum precursor, wait for the valve to open (clicking noise), and start timing. After 7–10 s, close the valve by clicking *Closed* again.

k. Click the *Insert* checkbox to retract the GIS needle.

l. In the Odemis graphical user interface, move the stage down by clicking the *Z-*button three times.

m. Once the *Coating* button becomes green (active) in the Odemis graphical user interface, click *Done* to close the *Move sample stage* window.

n. Click *3Beams* to move the sample positioning stage back and wait for the move to finish.

o. Switch to the *Alignment tab* of the Odemis graphical user interface and press *Move to latest custom alignment*.

p. Switch to the *Localization tab*.

4. Align FIB and SEM (10 min).

a. Enable the *RLMAcq* stream.

b. On the SEM overview image, look for a location with features that are visible in both SEM, FIB, and LM. In the *RLMAcq* stream, click on the spot to center and focus on the grid foil using the right mouse button.

c. In xT Microscope Control, apply the settings in Table 1 for the FIB and SEM.

**Table 1.** Settings for FIB and SEM

Image modality	Acceleration voltage (kV)	Current (pA)	Scanning resolution	Dwell (μs)	HFW (μm)	WD (mm)
SEM	1	25	1,536 × 1,024	1	600	4.4646
FIB	30	80	1,536 × 1,024	0.1	150	13.0

d. In xT Microscope Control, acquire two images, focus, and find corresponding features in both images, as in Figure 3 under Beam Alignment 1, Coarse.

e. Determine how the sample stage should be moved along Z to get the same feature centered in both the SEM and FIB FOV. To move the stage in a determined direction, run an RLM stream in the Odemis graphical user interface and adjust the focus.

f. To improve the alignment, you can increase the magnification in FIB and SEM and repeat B4d–e.

5. Align FIB and LM (15 min).

a. Open a Terminal prompt. While the RLM stream is running, focus the RLM using the following command:

odemis-cli --move align z 5

Here, the number 5 indicates the number of μm by which the objective lens will move, as well as the direction (positive). Adjust the number and/or sign and rerun the command until you are satisfied with the focus of the RLM.

b. Center the RLM stream at the feature used for FIB-SEM alignment using the following commands:

odemis-cli --move align x 5

odemis-cli --move align y 5

As in B5a, adjust the number and/or sign and rerun the commands until you are satisfied with the center of the RLM.

c. Move to a flat region on the grid.

d. Focus the RLM by moving the stage.

*Note: Do not use the Terminal prompt, as this will move the objective rather than the stage.*

e. Change the FIB current to 2.5 nA in xT Microscope Control and focus.

f. Mill a cleaning cross section of 2 × 2 μm centered in x and with the pattern top exactly at the center along the y-axis of the FIB FOV in xT Microscope Control. See Figure 3 under Beam Alignment 1, Fine.

g. Use the commands in the Terminal prompt to move the objective lens such that the alignment pattern, visible in the RLM (Odemis), is centered in x. The center of the FOV along y should be aligned with the damaged/undamaged foil edge.

h. Move to a different location on the foil and repeat steps B5d–g. If the alignment is good, you do not need to move the objective lens much at step B5g, and you can stop.

6. Check and refine targets for lamella fabrication (30 min).

a. In the *Localization tab* of the Odemis graphical user interface, enable the *RLMAcq* stream.

b. For each feature in the SEM overview image, do the following:

i. Click *Create/Move* button.

ii. Select the feature and drag it to the center of the grid square.

iii. Bring the foil into focus and click the *Use current Z* button to update the Z position of the feature.

iv. If you see crystalline ice on the backside of the grid (Figure 3 under Target check and refinement 1) or a broken grid foil, set the *Status* to *Discarded* in the dropdown menu.

v. Select the next feature in the dropdown menu under *Features.*

c. Switch to a fluorescent stream of your choice.

d. For each feature in the SEM overview image with status *Active*, do the following:

i. Click *Create/Move* button.

ii. Select the feature and drag it to the fluorescent feature you want to target.

*Note: For the feature to be accessible to both FIB and TEM tomography, it needs to be in the middle 40 μm of the grid square.*

iii. Bring the fluorescent feature into focus and click the *Use current Z* button to update the Z position of the feature.

iv. If there is no fluorescent feature in the grid square that you wish to mill, set the *Status* to *Discarded* in the dropdown menu.

v. Select the next feature in the dropdown menu under *Features.*

7. Automated rough milling (6 min/feature + 1 h to check and clean lamellae).

a. Check that the iFast script for automated milling is running:

i. Open the iFast developer.

ii. Click *open*.

iii. Navigate to the iFast script *lamella milling*.

iv. Click the run icon at the top.

b. In the Odemis graphical user interface, select *Milling* → *Auto mill* → *Relief cuts & rough milling* → *Run action*.

c. Wait for the rough milling to finish. Depending on the number of targets selected, this can take around 1 h.

d. Check all *Active* features using FM and discard targets that have lost the fluorescent signal and broken lamellae, such as the one shown in Figure 3 under Target check and refinement 2.

e. In xT Microscope Control, clean all *Active* lamellae with the FIB at 30 kV, 2.5 nA.

8. Automated lamella thinning (2 μm) (6 min/feature + 1 h to check and clean lamellae).

*Note: Thinning can also be done by hand as described in the next section, except with a FIB current of 0.79 nA.*

a. Decrease the FIB current to 0.79 nA and bring into focus.

b. Align the FIB and LM as described in step B5, except with a FIB current of 0.79 nA.

c. For each feature, bring the fluorescent target into focus and update the Z position. Set the status to *Active*.

d. In the Odemis graphical user interface, select *Milling* → *Auto mill* → *2 μm milling* → *Run action*.

e. Wait for the thinning to finish.

f. Check all *Active* features using RLM and discard broken lamella.

g. Check all *Active* features using FM and discard targets that have lost the fluorescent signal, such as the one shown in Figure 3 under Target check 3.

9. Thin lamella further by hand (15 min/feature).

a. Bring a fluorescent feature in the lamella into focus in the FM and center the crosshair on the feature. Keep the fluorescent stream running.

b. Decrease the FIB current to 0.23 nA and bring into focus.

c. To keep track of the fluorescence at the crosshair while milling, you can use the Jupyter notebook IFM-Monitor.ipynb (Figure 4A). Open the terminal and type jupyter-notebook. After hitting enter, a browser tab will open with a folder tree. Navigate to IFM-Monitor.ipynb, open the script, and run it. A live plot will appear with the maximum (orange) and mean (blue) pixel intensity around the crosshair in the FM image. Check if the fluorescent stream is live.

d. Draw a cleaning cross-section. Thin the lamella while tracking the fluorescence intensity. Immediately stop milling if the intensity drops rapidly. Continue milling from the other side.

*Notes:*

*1. The platinum layer is visible in RLM as a bright white band at the bottom of the lamella (Figure 4B, C) [10]. If the platinum layer is gone, you must stop milling, even if your lamella is not sufficiently thin yet.*

*2. Always pause the FIB before switching between light imaging channels, as the filter wheel induces vibrations.*

*3. Always pause the FIB before stage movements (e.g., focusing) and acquire a new FIB overview before you continue milling.*

e. To check the lamella thickness, measure the width of the interference pattern at the top edge of the lamella in RLM and use the equation *d = 0.174w*, where *d* is the lamella thickness, and *w* is the width of the interference pattern (Figure 4B, C) [10]. You can measure using the ruler icon on the left.

10. Polish lamella (5 min).

a. Decrease the FIB current to 80 pA.

b. Mill the lamellae to get an even surface. Intensity variations of the lamella visible in the RLM indicate variations in thickness [10]. Therefore, aim to get an even intensity of the lamella in RLM.

11. Acquire images before unloading (5 min).

a. In the Odemis graphical user interface, go to *Milling* → *Auto mill* → *Relief cuts & rough milling* → *Acq imags*.

b. Center on the grid and acquire a final SEM overview image.

c. Click *File* → *Save Snapshot As* to save the SEM overview image.

**C. Sample unloading**

1. Prepare the ENZEL (2 min).

a. Start the turbo molecular pump.

b. Check that the load-lock chamber is pumped by the roughing pump and the pressure is below 0.1 mbar.

c. When the turbo is above 50%, close the three-way valve and open the gate valve between the turbo-molecular pump and the load-lock chamber.

d. In the *Chamber tab* of the Odemis graphical user interface, click *Loading* to move the sample positioning stage to the loading position.

2. Prepare the glovebox (5 min). See Section A3.

3. Load cold table in transfer module (3 min). See Section A5.

4. Load cold table in ENZEL (5 min). See Section A6.

5. Unload shuttle in ENZEL (2 min).

a. Unscrew the transfer rod tip from the cold table.

b. Lower the cold table slightly.

c. Insert the transfer rod 30 cm while keeping it level.

d. Lower the end of the transfer rod to lift the tip of the rod and insert a bit further. The rod should be in the field of view of the Debian infrared CCD camera.

e. Attach the torque limiter at the end of the transfer rod.

f. Carefully insert the transfer rod further, aiming for the screw hole. Rotate the torque limiter end to screw the transfer rod tip into the shuttle. When the torque limiter clicks, the transfer rod is fully engaged in the shuttle.

g. Carefully remove the shuttle. Carefully retract the transfer rod 30 cm while moving your hand down slightly to lift up the front end of the rod.

h. Level the transfer rod and retract until the shuttle is inside the load-lock chamber.

i. Close the gate valve to the SEM chamber and secure the rest hook.

j. Lift up the cold table and carefully insert the shuttle into the cold table.

k. Unscrew the transfer rod from the shuttle.

6. Unload the cold table in ENZEL (3 min). See Section A8.

7. Unload cold table in glovebox (5 min). See Section A9.

8. Unmount the sample in glovebox (5 min).

a. Take the tools (fine tweezers, carbon-tipped tweezers, gridbox pen, and hex key) from the hotplate and slot them in the cold table to precool their tips. Position the large tweezers in the sample preparation bath. Wait for the tools to thermalize.

b. Open the shuttle cover with the large tweezers.

c. Use the hex key to remove the screw inside the shuttle and place it in an empty recess on the cold table.

d. Use the fine tweezers to take out the AutoGrid and place it in the second recess.

e. Take the Falcon tube with your gridbox from the storage dewar and place in the metal tea sieve basket inside the bath.

f. Place the gridbox into the fitting recess in the cold table using the big tweezer. Remove the lid with the gridbox pen.

g. Use the carbon-tipped tweezers to place the AutoGrid in the gridbox.

h. Re-cap the gridbox with the gridbox pen and place it into the Falcon tube. Place the Falcon tube into the storage dewar.

i. Pick up the spacer ring and place it inside the shuttle using the fine tweezers so that the flat side of the AutoGrid is facing upward.

j. Insert one fine tweezer tip into one of the small holes in the spacer ring and place the spacer ring in the shuttle with the opening facing the FIB access hole.

k. Use the hex key to place the screw on top of the shuttle and carefully screw it hand-tight.

l. Check that the top of the screw is below the shuttle surface. If this is the case, all parts are mounted correctly.

m. Close the shuttle cover.

n. Place all tools on the hot plate set to 70 °C and turn on the hot plate timer (4 h).

9. Warm up the microcooler and end-of-day checks (5 min).

*Note: Only follow these instructions if you do not plan to load any more samples today.*

a. Stop the cooling cycle by clicking *Stop temperature control* in CryoVision. Click the button once more to confirm.

b. Check the *Bottle level* in CryoVision and replace the gas bottle if the level is below 15%.

c. In xT Microscope Control, press *Sleep* (under *System*) to shut down the FIB-SEM.

d. Close the three-way valve and open the gate valve so that the load-lock chamber is pumped by the turbo molecular pump.

e. If you want to continue the next day, top up the small dewar mounted at the back of the chamber with liquid nitrogen.

## Validation of protocol

TEM images of the lamellae depicted in Figures 3 and 4 are included in Figure S3. We acquired a tomogram of an autolysosome (Figure S4 and Video S1). All TEM images were acquired on a JEOL JEM3200-FSC operated at 300 kV. For the montage in Figure S3A and B, 6 × 6 images at 2,500× magnification (1.152 nm per pixel) were stitched together using PySerialEM [19]. For the tomogram in Figure S4 and Video S1, MotionCor2 was used to motion-correct the movies [20]. Tilt-series alignment and tomogram reconstruction were performed with the IMOD tomographic package [21]. Tomograms were reconstructed with WBP and with a 10-iteration of the SIRT-like filter. The tomogram was denoised using TOPAZ-Denoise. The movie was made with ChimeraX [22].

Using the ENZEL system, Wang et al. [15] were able to investigate the NLRP3 inflammasome in situ.

This protocol or parts of it has been used and validated in the following research articles:

• Figure 6 in Boltje et al. [1]. A cryogenic, coincident fluorescence, electron, and ion beam microscope. *eLife*, 11, e82891. https://doi.org/10.7554/eLife.82891

• Figure 7 in Boltje et al. [10]. Thickness- and quality-controlled fabrication of fluorescence-targeted frozen-hydrated lamellae. *Cell Reports Methods*, 5(3), 101004. https://doi.org/10.1016/j.crmeth.2025.101004

## General notes and troubleshooting

**General notes**

1. Some training is required to perform the protocol. Loading and unloading the grid in the shuttle requires precise handling of the grid and shuttle components. To (un)load the cold table in ENZEL, many steps are required to be executed quickly and in the right order. If the user has experience with using a FIB, the milling process is straightforward and can be completed by following the protocol step by step.

2. Whenever you change the FIB current, you have to check the focus and adjust it if necessary.

3. If you move the objective, the features do not move along with it, as their position is stored with respect to the sample stage. Therefore, it is essential that you fine-tune the feature position after alignment.

4. In the process of milling lamellae, the FIB current is stepwise decreased. A high FIB current allows for faster ablation of material. However, high FIB currents result in a wider ion beam profile and more beam-induced damage. Therefore, the FIB current should be decreased as the lamella is thinned. We found that the FIB settings described in this protocol balance speed and accuracy. Users can adjust the FIB settings during lamella milling and thinning based on their needs.

5. It is important to regularly check the Pt coat and to stop when the coat is gone. One can easily and unintentionally ablate a large portion of the lamella once the Pt runs out.

6. Always pause the FIB before adjusting light imaging settings in Odemis to prevent milling in undesired locations. When you switch between imaging channels, the filter wheel induces vibrations. When you focus the RLM or move the crosshair, you move the stage.

7. Always acquire a new FIB overview when you want to continue milling, so that you are completely certain that you are actually milling in the position you anticipated.

**Troubleshooting**

Problem 1: When loading the cold table into ENZEL, you pumped too quickly; as a result, some liquid nitrogen solidified.

Solution: Open the roughing pump fully and wait until the loadlock chamber is evacuated to 0.1–0.2 mbar. This can take several minutes.

Problem 2: When (un)loading the shuttle in ENZEL, you displaced the stage with the transfer rod.

Solution: Retrieve the transfer rod and close the gate valve. In the *Chamber tab* of the Odemis graphical user interface, check the color of the *Loading* button. If it is green, click *3Beams*. Immediately *Cancel* and click *Loading.* If the *Loading* button is not green, click on the button. Wait until the stage stops moving. It is now again in the correct position to retry (un)loading the shuttle.

Problem 3: During alignment, you notice that the FIB view is blocked.

Possible cause: The sample is not loaded properly or not flat.

Solution: Unload the sample. Check if the coverslip in the shuttle is intact and seated in the right position. Check if the grid is flat.

Problem 4: You are unable to mill material in a certain region of the grid.

Possible cause: The coverslip is broken.

Solution: Unload the sample and replace the coverslip.

Problem 5: After automated milling, the holes above/below the lamella and/or the relief cuts are not fully milled through.

Possible cause: There was crystalline ice contamination on the top (cell side) or bottom of the grid.

Solution: Only select features for rough milling where no crystalline ice is visible in RLM and SEM.

Possible cause: There is too much vitrified water on the grid.

Solution: Consider your workflow for preparing grids with vitrified cells. You may want to increase the blotting time.

Problem 6: The fluorescence has disappeared completely after automated milling.

Possible cause: The FIB-LM alignment is off.

Solution: Take time to perform the FIB-LM alignment.

Possible cause: The fluorescent feature was not in focus at the stored Z position.

Solution: Take time to focus the fluorescent feature. Remember to select the feature and click the *Use current Z button* to update the Z position of the feature.

Problem 7: You run out of time to complete the preparation of the lamellae today.

Solution: You can unload the shuttle, but leave the sample inside the shuttle. Store the sample with shuttle inside the storage dewar and slide the shuttle back into the cold table next day. You can now reload the grid with the same orientation. This is not ideal, as the additional loading and unloading of the grid can result in more contamination.

## Supplementary information

The following supporting information can be downloaded here:

1. Figure S1. Custom interface between transfer module and glovebox

2. Figure S2. Assembly of custom interface between transfer module and glovebox

3. Figure S3. TEM images of lamella depicted in Figure 3 and Figure 4

4. Figure S4. Tomogram of autolysosome acquired from the lamella depicted in Figure 3

5. Video S1. Tomogram of autolysosome acquired from the lamella depicted in Figure 3

6. Table S1. Bill of materials for assembly to interface transfer slusher with glovebox
